# Where the light lingers

**DOI:** 10.1017/S1478951525101053

**Published:** 2025-10-24

**Authors:** Miguel Julião

**Affiliations:** Cuidados Paliativos, Equipa Comunitária de Suporte em Cuidados Paliativos PalCo, Amadora, Portugal

Love did not end
when your hand let go –
it paused,
like a candle flickering in wind,
uncertain
if it should die or blaze.
Death came quietly,
no grand trumpet,
just the hush of breath
folding into silence.
Even the walls listened.
I spoke your name
to the air –
not expecting an answer,
but because the sound
still held you.
Love is cruel like that.
It stays.
It sleeps in your sweaters,
sits with me in empty chairs,
calls me to bed
then rolls over to where you’re not.
Some nights,
death feels like a locked door
with light beneath it.
Love, the key
I don’t know how to turn.
Still,
I carry you –
in poems,
in morning coffee,
in the soft ache
of remembering without drowning.
Because even death
cannot undo
what love
insisted on becoming.

